# Tumour cell blebbing and extracellular vesicle shedding: key role of matrikines and ribosomal protein SA

**DOI:** 10.1038/s41416-019-0382-0

**Published:** 2019-02-11

**Authors:** Bertrand Brassart, Jordan Da Silva, Mélissa Donet, Emeline Seurat, Frédéric Hague, Christine Terryn, Fréderic Velard, Jean Michel, Halima Ouadid-Ahidouch, Jean-Claude Monboisse, Aleksander Hinek, François-Xavier Maquart, Laurent Ramont, Sylvie Brassart-Pasco

**Affiliations:** 1 Université de Reims Champagne Ardenne, SFR CAP-Santé (FED 4231), Laboratoire de Biochimie Médicale et Biologie Moléculaire, Reims, France; 2 CNRS UMR 7369, Matrice Extracellulaire et Dynamique Cellulaire - MEDyC, Reims, France; 3 Université de Picardie Jules Verne, SFR CAP-Santé (FED 4231), Laboratoire de Physiologie Cellulaire et Moléculaire, EA 4667 Amiens, France; 4 Plate-forme Imagerie Cellulaire et Tissulaire, Université de Reims Champagne-Ardenne, Reims, France; 5 Biomatériaux & inflammation en site osseux, EA 4691, SFR CAP-Santé (FED 4231), Université de Reims-Champagne-Ardenne, Reims, France; 6 Laboratoire de Recherche en Nanosciences, EA 4682, SFR CAP-Santé (FED 4231), Université de Reims-Champagne-Ardenne, Reims, France; 7 CHU Reims, Laboratoire Central de Biochimie, Reims, France; 8 Physiology & Experimental Medicine Program, Hospital for Sick Children, Toronto, ON Canada; 9 Institute of Medical Science, University of Toronto, Toronto, ON Canada; 10 Department of Laboratory Medicine and Pathobiology, University of Toronto, Toronto, ON Canada

**Keywords:** Extracellular matrix, Cancer microenvironment, Metastasis

## Abstract

**Background:**

Carcinogenesis occurs in elastin-rich tissues and leads to local inflammation and elastolytic proteinase release. This contributes to bioactive matrix fragment (Matrikine) accumulation like elastin degradation products (EDP) stimulating tumour cell invasive and metastatic properties. We previously demonstrate that EDPs exert protumoural activities through Hsp90 secretion to stabilised extracellular proteinases.

**Methods:**

EDP influence on cancer cell blebbing and extracellular vesicle shedding were examined with a videomicroscope coupled with confocal Yokogawa spinning disk, by transmission electron microscopy, scanning electron microscopy and confocal microscopy. The ribosomal protein SA (RPSA) elastin receptor was identified after affinity chromatography by western blotting and cell immunolocalisation. mRNA expression was studied using real-time PCR. SiRNA were used to confirm the essential role of RPSA.

**Results:**

We demonstrate that extracellular matrix degradation products like EDPs induce tumour amoeboid phenotype with cell membrane blebbing and shedding of extracellular vesicle containing Hsp90 and proteinases in the extracellular space. EDPs influence intracellular calcium influx and cytoskeleton reorganisation. Among matrikines, VGVAPG and AGVPGLGVG peptides reproduced EDP effects through RPSA binding.

**Conclusions:**

Our data suggests that matrikines induce cancer cell blebbing and extracellular vesicle release through RPSA binding, favouring dissemination, cell-to-cell communication and growth of cancer cells in metastatic sites.

## Introduction

Local invasion and metastasis of most malignant tumours require secretion of proteinases that degrade local extracellular matrix (ECM). This process causes accumulation of ECM degradation products. These products then interact with cell receptors and regulate tumour invasion and metastatic dissemination.^1,2^ Elastin is the major component of elastic fibres, abundant in blood vessels and lungs, but also present in the frameworks of other connective tissues and epithelial organs. Meaningfully, the partial proteolytic degradation of elastic fibres by several elastases causes formation of EDPs (a category of matrikines^3^), revealing cryptic sites that might potentially interact with cell receptors. EDPs have been blamed for their contribution to several pathologies like tumour progression and metastasis.^4–6^ EDPs promote the invasive and metastatic properties of tumour cells. Among EDPs, VGVAPG and AGVPGLGVG peptides were reported to stimulate cell invasion.^4–7^ They increase MMP-2, MT1-MMP, uPA and Hsp90 secretion by cancer cells. Hsp90 was shown to interact with many extracellular proteins involved in cancer progression and metastasis and to be of critical importance.^8^ Extracellular Hsp90 is associated with increased tumour invasiveness. Hsp90 increases MMP-2 stability and enzymatic activity.^9^ Hostile environmental conditions such as serum starvation, hypoxia and oxidative stress promote Hsp90 extracellular location through extracellular vesicles (EVs),^10^ like exosomes and ectosomes.

Five cell surface receptors mediate EDP effects: (i) the elastin receptor complex (ERC) [elastin-binding protein/neuraminidase-1/protective protein cathepsin A;^11^ (ii) αvβ3 integrin;^12^ (iii) αvβ5 integrin;^13^ (iv) galectin-3; and (v) ribosomal protein SA (RPSA).^14^ Expressed at all stages, RPSA was shown to exert many physiological roles. The RPSA was originally identified as a 37-kDa/67-kDa binding protein for laminin but is also a membrane receptor for growth factors, prion and pathogens.^15,16^ It contributes to the crossing of the blood–brain barrier by neurotropic viruses and bacteria. Contributing to cell receptor, RPSA also regulates ribosome biogenesis, cytoskeletal organisation, and nuclear functions.^17^ It governs critical cellular processes including growth, survival, migration, protein synthesis, development, and differentiation. RPSA has been associated with neurodegenerative diseases and developmental abnormalities and is a biomarker of metastasis.^18–23^

Proteinase requirement for the migration of highly invasive subpopulations of tumour cells is called into question. Results of clinical trials using proteinase inhibitors in cancers were disappointing.^24^ Authors identified a nonproteolytic-dependent mechanism of tumour invasion through ECM.^25^ Cancer cells from colon and squamous carcinomas display an elongated morphology in three-dimensional Matrigel matrix.^25^ By contrast, some melanoma and colon carcinoma cells display a rounded morphology in Matrigel matrix and their invasion is independent of extracellular proteinases. HT-1080 and MDA-MB-231 cells exhibit different plasticity, switching between these distinct modes of invasion. Sustained proteinase-independent migration results from a flexible amoeba-like shape change.^26^ Inhibiting protease activity reduces blebbing phenotype and promotes mesenchymal mode of HT-1080 and MDA-MB-231 cell migration.^27^ Elongated or mesenchymal modes of migration rely on actin polymerisation to push the plasma membrane forward. Amoeboid or rounded migration is highly dependent on ezrin/moesin/radixin (ERM)-controlled blebbing and actomyosin contractility.^28^ The mechanical aspects of amoeboid migration modes depend on hydrostatic pressure generated by calpain-dependent cytoskeletal protein degradation and actomyosin contractility.^29^ Besides the amoeboid migration movement, membrane blebbing allows extracellular vesicle shedding.^30^ Blebbing is RhoA-, Rho kinase (ROCK)-, and myosin light chain kinase (MLCK)-dependent.^28^ Clinical studies demonstrated their presence in different body fluids, and their potential as markers for the diagnosis, prognosis and surveillance of some diseases.^30^ EVs are classified into several categories according to their sise and origin: exosomes (30–100 nm diameter), microvesicles (100–1000 nm diameter), and a recently identified cancer-derived EV population termed “oncosomes”.^31^ Exosomes are either released from the cell when multivesicular bodies fuse with the plasma membrane or released directly from the plasma membrane. Microvesicles or oncosomes are formed by outward blebbing from the plasma membrane and are then released by proteolytic cleavage from the cell surface.^32,33^ Extracellular vesicles contain bioactive molecules, mRNA, miRNA and many proteins such as Hsp90 and proteinases.^34^ Extracellular vesicles shed from tumour cell lines facilitate proliferation, angiogenesis and ECM invasion.^35,36^

In this paper, we prove that EDPs induced blebbing, extracellular vesicle shedding and cell invasion. We also determined the involved receptor and molecular mechanisms.

## Methods

### Reagents

Synthetic elastin peptides (VGVAPG, AGVPGLGVG, GRKRK and TAMRA-AGVPGLGVG) were purchased from Proteogenix. Mouse anti-MMP-2 and anti-uPA antibodies were from Calbiochem. Y27632, blebbistatin, U0126, PD150606, lactose, chondroitin sulphate, nifedipine and EDTA were from Sigma-Aldrich. EGCG was purchased from Enzo Life Sciences. Rabbit anti-Hsp90, anti-cleaved caspase-3, anti-integrin αV, anti-p-ERM, mouse anti-αvβ3 and anti-αvβ5 integrin antibodies were from Ozyme. Rabbit anti-RPSA, anti-MMP-14, anti-calpain1, anti-ROCK2, anti-myosin light chain kinase, mouse anti-RPSA, anti-RhoA, anti-ROCK1 and mouse IgM isotype control antibodies were purchased from Abcam. Rabbit anti-p-LIMK-and goat anti-cofilin and anti-actin were from Santa Cruz. Anti-integrin αvβ3 antibody was purchased from Millipore. Annexin-5 alexa fluor^®^ 568, CellTrace Calcein Red-Orange, AM and DiOC18^3^ were from Invitrogen.

### Materials

Insoluble elastin was prepared from bovine ligamentum nuchae by hot alkali treatment and its purity was assessed by amino acid analysis and lack of hexoses and hexosamines in the preparation. Soluble EDPs were obtained from purified insoluble elastin by organo-alkaline hydrolysis. Peptides with an average molecular weight of 75 kDa were isolated by gel permeation on Sephadex G100.

### Cell lines and expression constructs

Human fibrosarcoma cells (HT-1080), breast carcinoma cells (MDA-MB-231, MDA-MB-435), melanoma cells (SK-MEL-28, HT-144, A375), colon adenocarcinoma cells (HT-29), lung carcinoma cells (BZR) and hepatocarcinoma cells (HUH-7, PLC-PRF-5) were obtained from the ATCC. Primary human dermal fibroblasts (HDFs) were obtained from abdominal skin (Plastic Surgery Department, Reims Clinic), with the written informed consent of donors and approval by the local ethical committee according to the principles of the Declaration of Helsinki. Cells were cultured in DMEM (Gibco-BRL) supplemented with 10% foetal calf serum.

### Cell transfection

Cell transfections with plasmids or siRNA were performed as previously described.^5^ The pEGFP-Hsp90 plasmid was a gift from Dr. J. Kim (Korea University, South Korea), the mCherry-MLC plasmide was a gift from Dr. S. Hooper (Cancer Research UK London Research Institute). The expression of appropriate proteins was confirmed by western blotting. siRNA specific to human RPSA and negative control siRNA (non-targeting pool), which do not target any genes, were purchased from Qiagen. The siRNA target different regions of the RPSA mRNA: First siRNA target sequence (5′-AGG-CTC-TTA-AGC-AGC-ATG-GAA-3′), 2nd siRNA target sequence (5′-TAC-CTG-GGA-TTG-CAT-ATC-AAA -3′), 3rd siRNA target sequence (5′-TTG-CAT-ATC-AAA-GCA-TAA-TAA-3′) and 4th siRNA target sequence (5′-TCG-ACA-TGA-GTT-GTA-CTT-CTA-3′).

### Blebbing assay

Cancer cells were pre-incubated in DMEM without FCS in the presence or absence of RPSA-blocking monoclonal antibody (10 µg/mL), 10 µM U0126, 25 µM Y27632, 50 µM PD150606, 5 mM EDTA, 50 µM blebbistatin and 10 µM EGCG, and stimulated or not by adding EDPs (50 µg/mL). After incubation at +37°C, the cells were fixed with 1.1% glutaraldehyde at the indicated times. The number of blebbing cells was evaluated by counting under a Zeiss Axiovert25 microscope (10 fields/well).

### Isolation of extracellular vesicles from cell-conditioned medium

Extracellular vesicles were prepared from cell-conditioned medium as previously described.^36^ Briefly, after a 24 h incubation of subconfluent cells in the presence or absence of EDPs, FCS-free conditioned medium was centrifuged at 300 × *g* for 10 min and at 800 × *g* for 15 min. The supernatant was centrifuged at 100,000 × *g* for 1 h at +4 °C, and the pelleted EVs were resuspended in PBS.

### Preparation of EV and cell extracts

EVs were pelleted by centrifugation at 100,000 × *g* for 1 h at +4 °C, supernatants were discarded and proteins were extracted from the pellet using RIPA buffer. Cell layers were scrapped in RIPA buffer. Both lysates with the Halt™ Protease and Phosphatase Inhibitor Cocktail (Thermo Fisher Scientific, Illkirch, France) and scraped. Lysates were mechanically resuspended by pipetting and vortexing every 5 min for 30 min. They were then centrifuged at 10,000 × *g* for 10 min at +4 °C to discard insoluble protein and the amount of soluble protein was quantified by Bradford assay method (Bio-Rad) using bovine serum albumin (Sigma) as a standard.

### Ca^2+^ measurements

The cytosolic calcium concentration was measured using FURA-2-loaded HT-1080 cells. The glass coverslip was mounted in a chamber on a Zeiss microscope equipped for fluorescence (excitation wavelengths: 350–80 nm, emission wavelength: 510 nm). Acquisitions were performed using a Cool SNAP HQ camera (Princeton Instruments, Evry, France) and Metafluor software (version 7.1.7.0) was used for acquisition and analysis. The [Ca^2+^]_i_ concentration was derived from the ratio of the fluorescence intensities for each of the excitation wavelengths (F_350_/F_380_), and from the Grynkiewicz equation. The cells were continuously perfused with saline solution and EDPs were added as 50 µg/mL via a whole chamber perfusion system. The flow rate of the whole cell chamber perfusion system was set to 1 mL min^−1^ and the chamber volume was 500 mL.

### Collagen I and 3D-matrix preparation

Acid-extracted, non-pepsinised type I collagen from rat tail tendons was prepared as previously described.^37^ Collagen was prepared for 3D-matrix experiments at a final density of 1.5 mg mL^−1^ using a 1:1 mixture of type I rat tail collagen and serum-free DMEM containing EDPs (50 µg/mL) and/or blebbistatin (50 µM). A GFP-Hsp90 HT-1080 cell pellet was resuspended at a final concentration of 10^5^ cells mL^−1^ in the previous mixture and 1 mL was added to the bottom of a 35 mm dish (Biovalley). The collagen gels were formed by incubating (+37 °C, 5% CO_2_) for 15 min, then FCS-free containing media were added and cell/collagen constructs were allowed to incubate 24 h before beginning confocal imaging.

### Preparation of conditioned media and zymography analysis

Cells were grown to subconfluence in 24-well culture plates (VWR) in 10% FCS-containing medium. After 16 h of FCS deprivation, EDPs (50 µg/mL) were added to FCS-free culture medium and cells were incubated for 24 h. Conditioned media were harvested and centrifuged at 800 × *g* for 10 min at room temperature to remove cellular debris. Conditioned media from HT-1080 cell cultures were analysed for zymography as previously described.^5^

### Western blot

Western blot analysis were performed as previously described.^5^

### Primer design for RT-qPCR

RT-qPCR primers were designed according to sequence of RPSA (NM_002295). The forward primer for RPSA was 5′-CCA-TTG-AAA-ACC-CTG-CTG-AT-3′ and the reverse primer was 5′- CTG-CCT-GGA-TCT-GGT-TAG-TGA-3′ with a 144 bp product. The forward primer for EEF1a1 was 5′-CTG-GAG-CCA-AGT-GCT-AAC-ATG-CC-3′ and the reverse primer was 5′-CCG-GGT-TTG-AGA-ACA-CCA-GTC-3′ with a 221 bp product. All primers were synthesised by Eurogentec.

### RT-qPCR

Total RNA isolation, reverse transcription and real-time PCR analysis were performed as previously described.^5^

### Affinity chromatography

HT-1080 protein extracts were chromatographed at +4 °C on a HiTrap column (GE Healthcare) previously functionalised with VGVAPG3x or AGIPGLGVGVGVPGLGVGAGVPGLGVGAGVPGFGAG elastin peptides, according to the manufacturer instructions. VGVAPG3x and AGIPGLGVGVGVPGLGVGAGVPGLGVGAGVPGFGAG elastin peptides were used instead of VGVAPG and AGVPGLGVG peptides to avoid the accessibility problems of small peptides due to bead grafting. Unbound material was removed with 30 mL of washing buffer (10 mM Tris, 1 mM CaCl_2_, 1 mM MgCl_2_, pH 7.6, 1/100 ProteoBlock Protease Inhibitor Cocktail (Fermentas), 0.1% octylglucoside). Proteins bound to the affinity column were then eluted with elution buffer (10 mM Tris, pH 7.6, 1/100 PIC, 0.1% octylglucoside) supplemented with increasing concentrations of NaCl (0.15, 0.6 and 1 M). Eluted samples were then solubilised in SDS sample buffer with 10 mM DTT, denatured at +95 °C for 5 min and submitted to silver staining (Silver Stain Plus^TM^ BioRad) and western blot using RPSA antibodies.

### Confocal microscopy

Immunofluoresence analysis were performed as previously described.^5^

Collagen matrix was imaged using backward second harmonic generation (SHG) signal. This signal was collected using 860 nm excitation wavelength with 420–440 bandpass filters. Image acquisitions were performed with ZEN Software (Carl ZEISS SAS) and all acquisition settings were constant between specimens.

### Real-time imaging and analysis

For live imaging, the HT-1080, GFP-Hsp90 HT-1080 and mCherry-MLC HT-1080 cells were grown on µ-dish 35 mm and stimulated with 50 µg/mL EDPs. Cells were placed directly on a heated stage and supplemented with 5% CO_2_. Images were acquired using Axio Observer (Carl ZEISS SAS) coupled with confocal Yokogawa spinning disk (Roper Scientific, Evry, France) through a 63 × NA1.40 oil immersion objective. The system was drived by Metamorph Imaging software (Roper Scientific) and signals were collected with EM-CCD Camera EVOLVE (Photometrics). The whole fluorescent cell bodies were captured in Z-stack with 0.5 µm step between optical sections. Each Z-stacks were acquired in ∼22 s intervals during 20 min. The experimental results show representative cells from a minimum of 10 different experiments. Cell sections of the 2D real-time sequences were created using ImageJ plugin. Visualisation of 4D data was done using Imaris software (Bitplane AG).

### Scanning electron microscopy

Cells were rinsed twice with D-PBS (Gibco) and then fixed with 2.5% glutaraldehyde in D-PBS (Sigma-Aldrich) for 1 h at room temperature. After gradual dehydration in ethanol (50, 70, 90 and 100 twice), the samples were immersed in hexamethyldizilazane (Sigma-Aldrich) for 5 min, air dried at room temperature and finally sputtered with a thin gold–palladium film under a JEOL ion sputter JFC 1100. Cells were visualised using a LaB6 electron microscope (JEOL JSM-5400 LV, JEOL) at an acceleration voltage of 20 kV using the secondary electron signal.

### Transmission electron microscopy

Isolated extracellular vesicles were visualised at room temperature using a FEG electron microscope (JEOL2100F, JEOL) fitted with a CCD camera (Orius SC200D, Gatan) at an accelerating voltage of 200 kV.

### In vitro invasion assay

Invasion was performed as previously described.^5^

### Statistical analysis

Each experiment was performed at least thrice from separate sets of culture and data were expressed as mean ± S.E.M. Comparisons were performed using Student’s *t*-test. Results were considered significant when *p < 0.05, **p < 0.01, ***p < 0.001.

Correlation between different groups was analysed by using nonparametric statistical analysis. Spearman rank correlation coefficients (R) and Pearson correlation coefficients (P) were determined. *p*-values <0.05 were considered to indicate a statistically significant difference.

## Results

### Induction of cell surface blebbing by EDPs on cancer cells

**Fig. 1:**
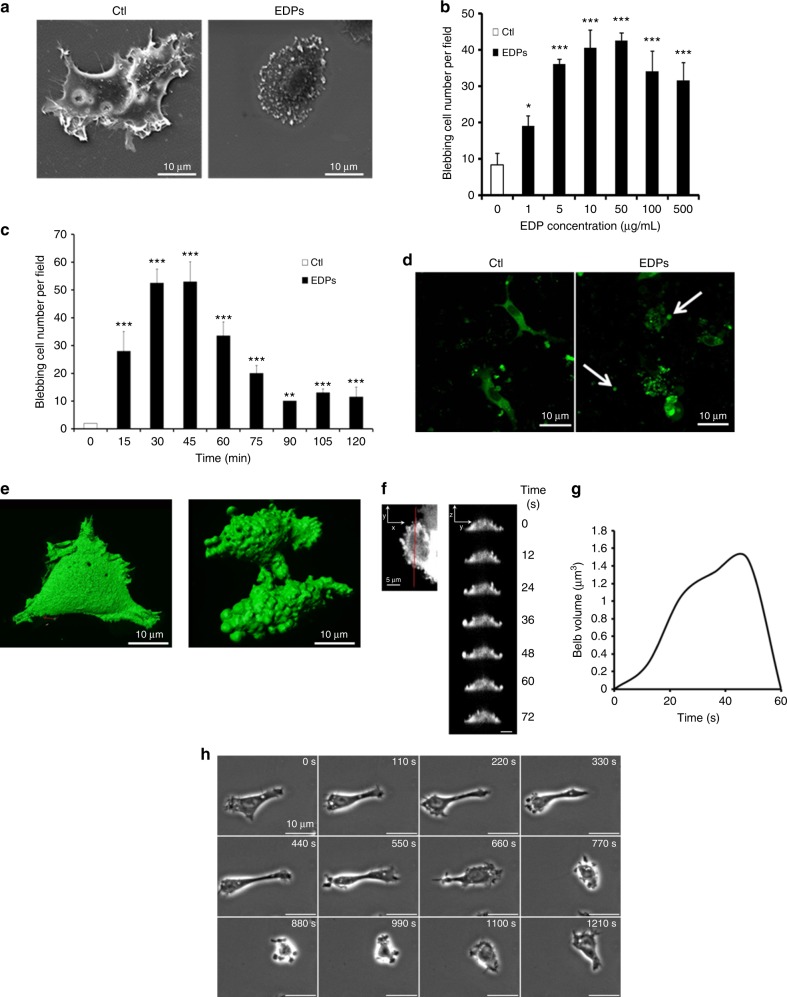
EDPs cause fast membrane bleb formation in HT-1080 cells. **a** Scanning electron microscopy of control (Ctl) and EDP-treated HT-1080 cells after 40 min of incubation. Scale bar: 10 µm. **b** EDP dose-dependent induction of blebbing was evaluated by counting 10 fields per well under a phase contrast optical microscope after a 40 min incubation. Results were expressed as mean ± S.E.M. Data from one experiment, representative of three independent experiments, are shown. **c** HT-1080 cells were treated with 50 µg/mL EDPs from 0 to 120 min. The quantification of the blebbing cell number was evaluated by counting 10 fields per well under a phase contrast optical microscope. Results were expressed as mean ± S.E.M. Data from one experiment, representative of three independent experiments, are shown. **d** EDPs stimulate cell membrane blebbing in 3D collagen matrix. GFP-Hsp90 transfected HT-1080 cells were seeded in a 3D collagen matrix in the presence of EDPs. 3D confocal imaging was realised after 24 h of incubation to study the HT-1080 cell morphology. In the presence of EDPs, HT-1080 cells adopted an amoeboid morphology with many membrane blebs and shed extracellular vesicles (white arrows). Scale bar: 10 µm. **e** Real-time imaging and analysis of bleb formation in GFP-Hsp90-transfected cell. Live-cell imaging by microscope coupled with confocal Yokogawa spinning disk of control and EDP-treated HT-1080 cells. **f** Analysis of bleb expansion and retraction on cell sections (red line *Y*-axis) on the 3D real-time sequences. Snapshots of the time series at 12 s intervals from which the analysis were generated (*Z*-axis/*Y*-axis). Data from one experiment, representative of 10 independent experiments are shown. **g** Graph of a bleb extension and retraction following the cell section analysis. Results are means of a representative experiment (*n* = 10) repeated on five independent experiments. **h** Snapshots of time-lapse phase-contrast microscopy movies of HT-1080 blebbing cell in presence of EDPs. Scale bar: 10 µm. *p < 0.05, **p < 0.01, ***p < 0.001, significantly different from control

During EDP treatments, cancer cell morphology was studied. Electron microscopy analyses revealed cell morphology modifications induced by EDP treatment. Without EDPs, HT-1080 cells presented a flat body with lamellipodia and filopodia (Fig. 1a). EDPs induced a cell surface blebbing characterised by a retracted cell body covered with blebs but devoid of lamellipodia and filipodia.

Cell surface blebbing was analysed by phase contrast microscopy in HT-1080 cell line with or without EDP treatment. Without EDPs, only few HT-1080 cells presented spontaneous cell surface blebbing. The number of blebbing cells increased dose-dependently from 1 to 50 µg/mL (Fig. 1b). At higher concentrations, the blebbing cell number appeared to decrease. EDPs (50 µg/mL) induced bleb formation as soon as 15 min after incubation (Fig. 1c). The maximum number of blebbing cells was obtained 30–45 min after EDP treatment.

Extracellular vesicles were reported to express high levels of Hsp90 protein.^38^ HT-1080 cells were stably transfected with GFP-Hsp90 recombinant protein. To study their morphology in a 3D model, cells were cultured for 24 h in a collagen matrix. In absence of EDPs, cells adopted a mesenchymal cell morphology (Fig. 1d; Suppl.figure 1). In presence of EDPs, HT-1080 cells adopted an amoeboid morphology with many membrane blebs and they shed extracellular vesicles (white arrows). To monitor EDP-dependent blebbing over time, GFP-Hsp90 HT-1080 cells were plated on coverslips and studied by spinning disk microscopy. Control cells presented a mesenchymal cell phenotype (Fig. 1d) (video 1; Suppl.video A). EDP treatment induced a cell surface blebbing (Fig. 1d) (video 2; Suppl.video B) and Hsp90 was detected in the blebs. Blebbing GFP-Hsp90 HT-1080 cell sections were performed. Attention was focused on the GFP-Hsp90 distribution along protrusions and on formation of membrane blebs (Fig. 1e–f). Snapshots of the time series (0–72 s) revealed a high GFP-Hsp90 concentration in the blebs. In most cells, the bleb volume grew smoothly during the first seconds (0.03 + 0.01 µm^3 ^s^−1^) to reach a maximum of 1.47 + 0.47 µm^3^ after 45 s and then decreased rapidly. Plasma membrane blebbing was highly dynamic in the presence of EDPs, with a bleb cycle lasting about 48 + 18 s. Bleb growth ceased and bleb retraction occurred with velocities of 0.12 + 0.02 µm^3^ s^−1^. EDP-induced bleb formation occurred over the entire cell surface but with larger bleb volume on cell outline. Moreover, bleb disappearance was concomitant with bleb formation in another position.

**Fig. 2:**
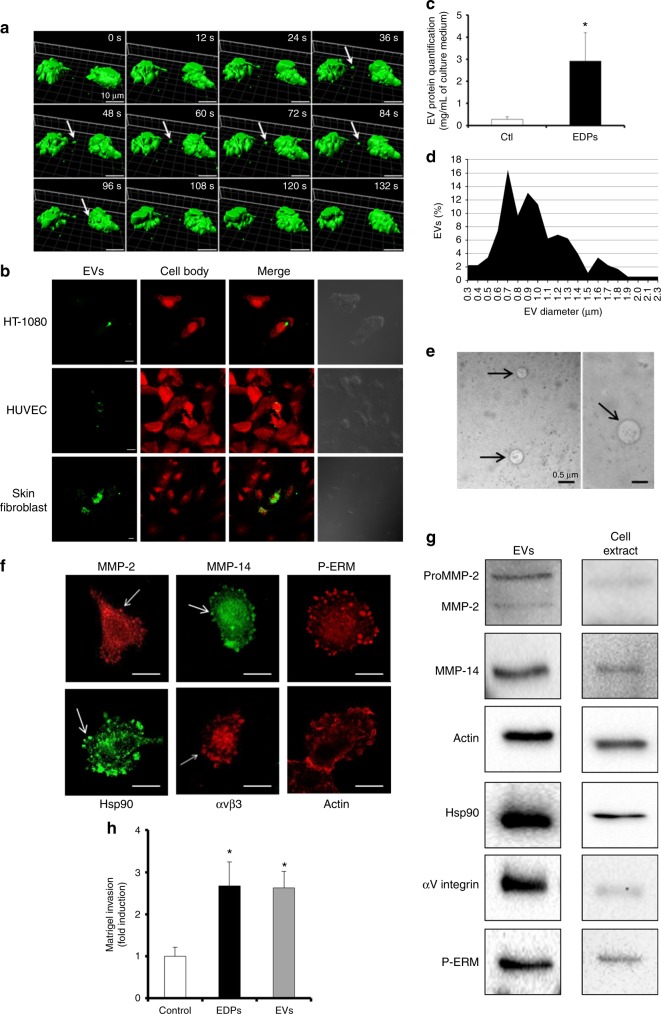
EDP-induced membrane blebbing leads to tumour extracellular vesicle shedding. **a** 3D Time-lapse snapshots of cell-to-cell communication between two blebbing cells in the presence of EDPs. Snapshot of 3D data was done using Imaris. White arrows indicate tumour shed extracellular vesicle. Scale bar: 10 µm. **b** Tumour extracellular vesicle binding and uptake by target cells. DiOC18(3) (green) stained EDP-dependent tumour shed extracellular vesicles were incubated for 6 h with modified calcein red-orange (red) staining tumour HT-1080 cells, HUVEC or human skin fibroblasts. Confocal imaging was realised to study the extracellular vesicle localisations. Scale bar: 10 µm **c** Extracellular vesicles were prepared from cell-conditioned medium by centrifugation and ultracentrifugation after 24 h of incubation. The extracellular vesicle (EV) protein content per mL of HT-1080 cell culture media in presence or absence of EDPs was measured by the Bradford micro-assay. Data are expressed as mean ± S.E.M. Values from five independent experiments, each performed in triplicate. *p < 0.05, significantly different from control. **d** Diameters of the isolated extracellular vesicles were measured by optical microscopy and expressed as a percentage of extracellular vesicles. **e** Structure of isolated extracellular vesicles (black arrows) visualised by transmission electron microscopy. The diameter of observed vesicles ranged from 300 to 700 nm. Scale bar: 0.5 µm. **f** Confocal microscopy analysis of MMP-2, MMP-14, P-ERM, Hsp90, αvβ3 integrin and actin in EDP-induced membrane blebs. White arrows indicate membrane blebs. Scale bar: 5 µm. **g** One microgram of extracellular vesicle (EV) proteins and 100 µg of cell extract proteins were analysed by western blot. **h** Quantification of Matrigel invasion by HT-1080 cells in presence of EDPs or extracellular vesicles (EVs) (amount of EVs corresponding to 1 µg of extracellular vesicle protein) after 6 h of incubation. Data are expressed as mean ± S.E.M. Values from three independent experiments, each performed in triplicate. *p < 0.05, significantly different from control

**Fig. 3:**
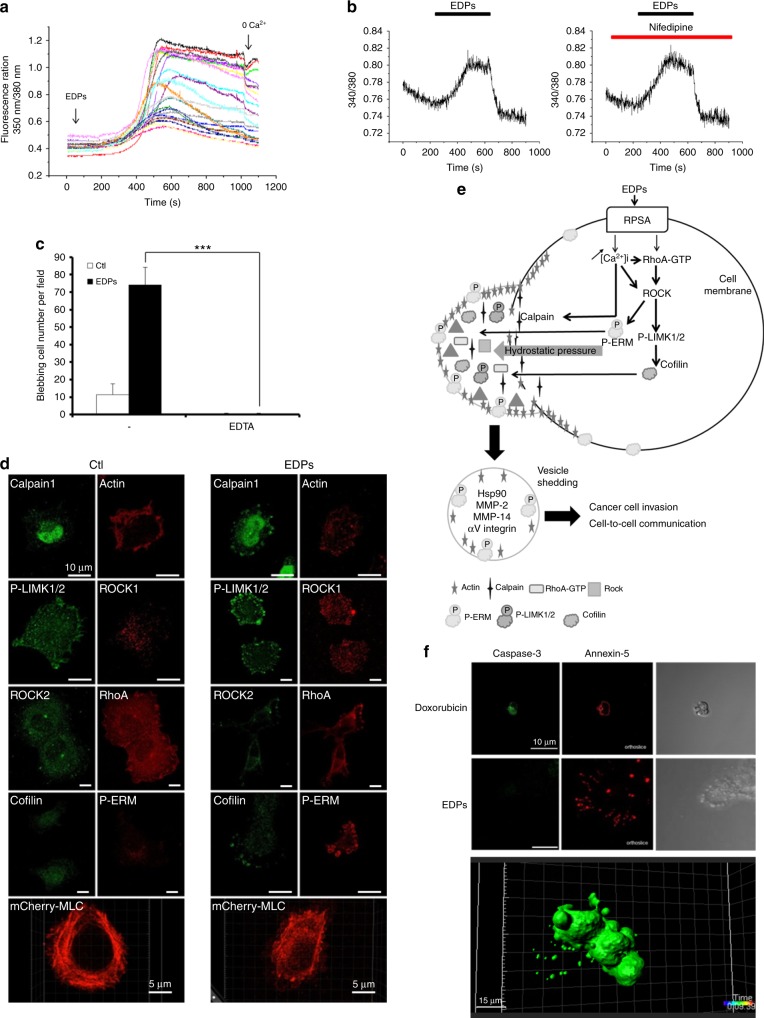
EDP-induced blebbing depends on calcium influx and on RhoA/ROCK/MLC signalling pathway. **a** Measure of cytosolic calcium concentration using FURA-2 loaded HT-1080 cells in response to EDP stimulations. Calcium flux is reported as ratio 350 nm/380 nm fluorescence of FURA-2 in twenty HT-1080 cells. Data from one experiment, representative of five experiments are shown. Calcium-free media (0 Ca) were used to confirm the key role of the extracellular calcium. **b** Measure of cytosolic calcium concentration using FURA-2 loaded HT-1080 cells in presence of nifedipine after stimulation with EDPs. Calcium flux is reported as ratio 340 nm/380 nm fluorescence of FURA-2 in HT-1080 cells. The results shown are representative experiments (*n* = 3). Nifedipine was used at 10 µM. **c** Blebbing inhibition in HT-1080 cells in the presence of EDTA, evaluated by counting 10 fields per well under a phase contrast optical microscope after EDP stimulation for 40 min. Data from one experiment, representative of three independent experiments, are shown. **d** Immunolocalisation by confocal microscopy of RhoA, ROCK2, ROCK1, P-LIMK1/2, cofilin, P-ERM, calpain1, actin and snapshot of mCherry-MLC transfected cells after treatment with EDPs versus untreated control HT-1080 cells (Ctl). Snapshot of 3D data was done using Imaris. Scale bar: 10 µm. **e** Schematic representation of the signalling pathways leading to EDP-induced blebbing and extracellular vesicle shedding**. f** Images from confocal microscopy analysis of cleaved caspase-3 and annexin-5 distributions after treatment with doxorubicin and EDPs. Cells were cultured on glass slides, fixed with paraformaldehyde and labelled with an anti-cleaved caspase-3 antibody (green) and annexin5-AF568 (red). Scale bar: 10 µm

Snapshots of the time series (0–20 min) generated by living cell microscopy showed a dynamic and transient blebbing (Fig. 1g; video 3; Suppl.video C). In the presence of EDPs, HT-1080 cells acquired an amoeboid morphology, and returned to a spreading morphology within 770 s. This result shows the reversibility of the blebbing. At lower magnification, after adding EDPs to the culture medium, HT-1080 cells presented a blebbing phenotype and a rapid shed extracellular vesicle accumulation (Suppl.figure 2; Suppl.video D). To exclude any effect of EDPs on cell viability, trypan blue exclusion was performed. EDP treatment did not alter cell viability (Suppl.figure 3).

### EDP-induced membrane blebbing leads to tumour extracellular vesicle shedding

**Fig. 4:**
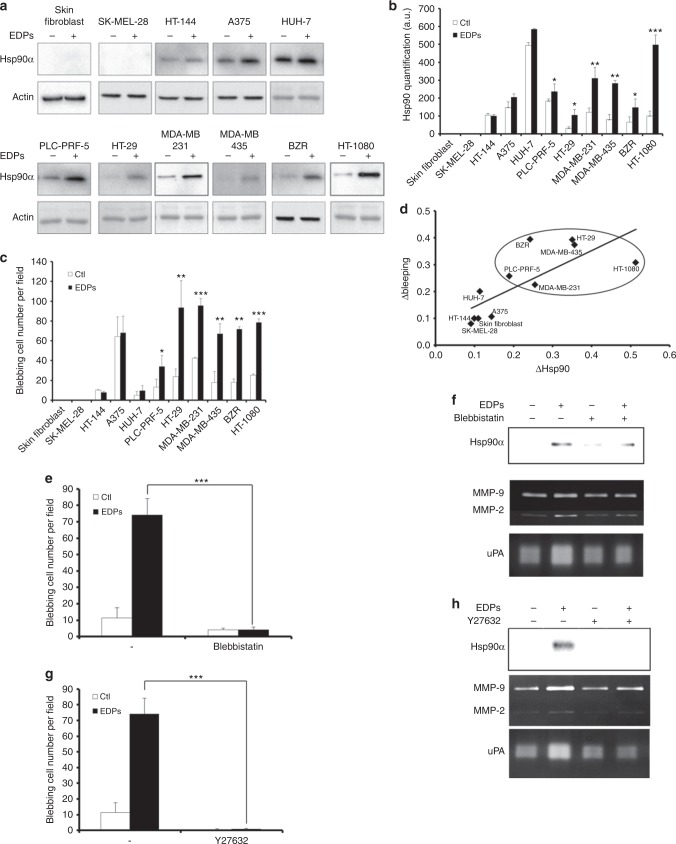
Correlation between EDP-stimulated Hsp90 secretion and EDP-induced membrane blebbing. **a** Different cell types were treated with EDPs and extracellular Hsp90 was analysed by western blot. Actin level attested the equal amount of protein loaded in the control and EDP. Data from one experiment, representative of three independent experiments, are shown. **b** Quantitative evaluation of Hsp90 secretion by different cell types. **c** Quantification of the blebbing cell number was evaluated by counting 10 fields per well under a phase contrast optical microscope after EDP stimulation for 40 min. Data from one experiment, representative of three independent experiments, are shown. **d** Correlation between Δblebbing and ΔHsp90 secretion in different cell types. Δblebbing = EDP-dependent blebbing – unstimulated blebbing. ΔHsp90 = EDP-dependent Hsp90 secretion – unstimulated Hsp90 secretion. Linear regression test was performed for each analysis and the R and *p* values are indicated below the graph. **e** Blebbing inhibition in HT-1080 cells in the presence of blebbistatin (50 µM), evaluated by counting 10 fields per well under a phase contrast optical microscope after EDP stimulation for 40 min. Data from one experiment, representative of three independent experiments, are shown. **f** HT-1080 cells were pretreated with blebbistatin (50 µM) for 1 h then stimulated by EDPs. Cell culture media were analysed for Hsp90, MMP-2, MMP-9 and uPA secretions (24 h of treatment) by western bot, gelatin zymography and gelatin-plasminogen zymography. **g** Blebbing inhibition in HT-1080 cells in the presence of Y27632 (25 µM), evaluated by counting 10 fields per well under a phase contrast optical microscope after EDP stimulation for 40 min. Data from one experiment, representative of three independent experiments, are shown. **h** HT-1080 cells were pretreated with Y27632 (25 µM) for 1 h then stimulated by EDPs. Cell culture media were analysed for Hsp90, MMP-2, MMP-9 and uPA secretions (24 h of treatment) by western bot, gelatin zymography and gelatin-plasminogen zymography

Snapshots of the time series (0–20 min) generated by living cell microscopy showed cell–cell communication via extracellular vesicles (Fig. 2a; video 4). In the presence of EDPs, HT-1080 cells rapidly presented blebbing which lead to extracellular vesicle shedding (white arrow, Fig. 2a). In some cases, extracellular vesicle binding to neighbouring blebbing cell resulted in fusion or endocytosis of the extracellular vesicle. This extracellular vesicle-dependent intercellular communication was also observed between a blebbing cell and a mesenchymal cell (Suppl.figure 4; Suppl.video E). Tumour extracellular vesicles bound to cancer cells and stromal cells like endothelial cells (HUVEC) and human skin fibroblasts (Fig. 2b). The green stained extracellular vesicles were internalised in some stromal cells. Extracellular vesicles were isolated from FCS-free media conditioned by HT-1080 cells incubated or not with EDPs for 24 h. In absence of EDPs, HT-1080 cells released 0.28 µg/mL of protein by the mean of extracellular vesicle proteins (Fig. 2c). Upon stimulation by EDPs, HT-1080 cells released 3.0 µg/mL of protein by the mean of extracellular vesicles. This increase could reflect either an increase in the number of released extracellular vesicles or an increase in the content of protein in extracellular following EPD treatment.

The extracellular vesicle diameters analysed by optic microscopy varied from 0.3 to 3.3 µm (Fig. 2d). Electron microscopy observations confirmed the heterogeneous sise of extracellular vesicles (Fig. 2e). Shed extracellular vesicles presented diameters similar to those of blebs.

**Fig. 5:**
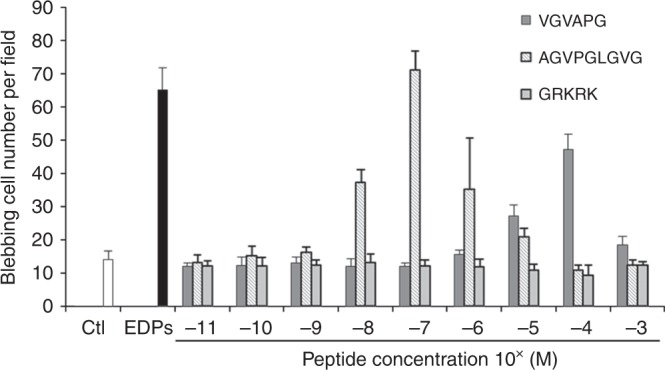
VGVAPG and AGVPGLGVG synthetic elastin peptides induced membrane bleb formation. HT-1080 cells were treated with three different synthetic peptides at concentrations ranging from 10^−11^ to 10^−3^ M for 40 min. The quantification of the blebbing cell number was evaluated by counting 10 fields per well under a phase contrast optical microscope. Results were expressed as mean ± S.E.M. Data from one experiment, representative of three independent experiments, are shown

We analysed different markers to confirm that extracellular vesicles originate from membrane blebs and from multivesicular bodies (Suppl.figure 5). These blebs contained large amounts of Hsp90, MMP-2, MMP-14, αvβ3 integrin, actin and P-ERM (Fig. 2f). The extracellular vesicle content was analysed. Proteins found in the blebs were also present in the extracellular vesicles: MMP-2 (pro- and active form), MMP-14, actin, Hsp90, αv integrin subunit and P-ERM (Fig. 2g). The presence of extracellular vesicles (EV amount corresponding to 1 µg of extracellular vesicle proteins) significantly stimulated the HT-1080 Matrigel invasion (Fig. 2h).

### EDP-induced blebbing depends on calcium influx and RhoA/ROCK/MLC signalling pathway

HT-1080 cells treated with EDPs for 5 min showed a strong increase in cytosolic calcium concentration [Ca^2+^]_i_ after a latency period of about 400 s (Fig. 3a). In these conditions, the suppression of extracellular calcium (0 Ca^2+^) led to a marked decrease in [Ca^2+^]_i_. Nifedipine did not inhibit EDP-dependent calcium flux, excluding a L-channel type role^39^ (Fig. 3b). In addition, treatment with EDTA, a chelating agent, completely repressed blebbing (Fig. 3c). Taken together, these results confirmed the key role of the extracellular calcium in the regulation of cell surface blebbing though unknown active channels.

**Fig. 6:**
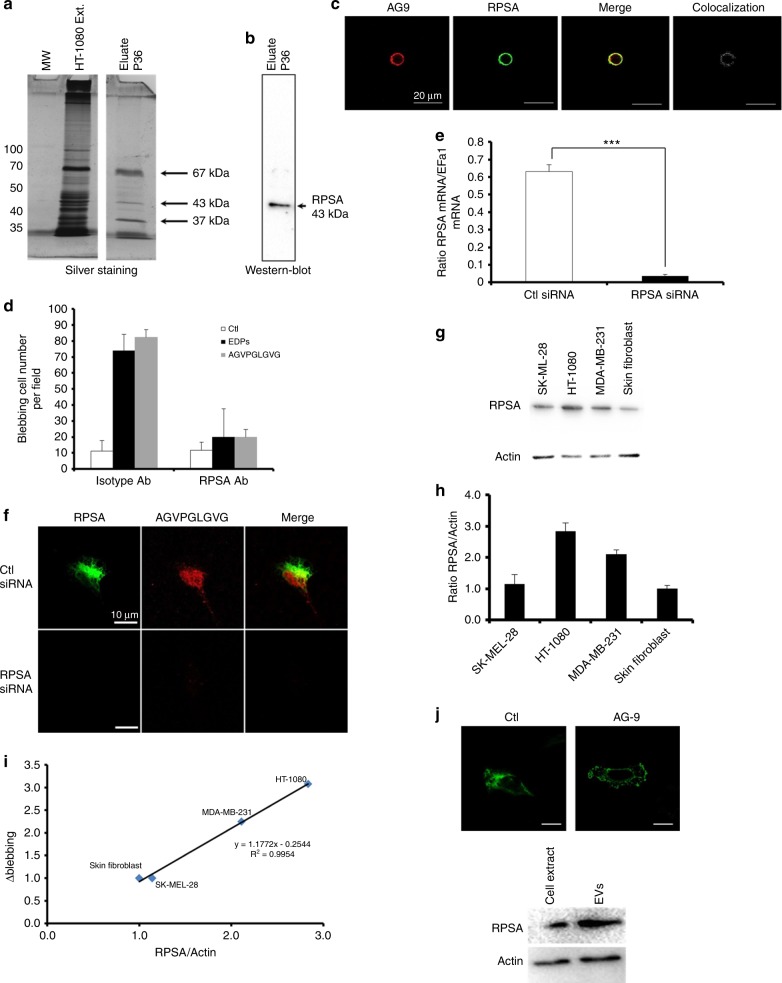
EDP-induced blebbing involves the RPSA elastin-laminin receptor. Identification of the RPSA protein as the EDP receptor by affinity chromatography. **a** Eluted samples were submitted to silver staining and **b** western blot using RPSA antibodies. **c** Optical section realised by confocal microscopy of HT-1080 cells incubated with Tamara-AG9 (AG9) at 5 × 10^−5^ M for 1 h at +4 °C and after immunolocalisation of RPSA protein. Colocalisations were studied with Colocalisation plugin of ImageJ. Scale bar: 20 µm. **d** Blebbing quantification in HT-1080 cells in the presence or not of RPSA-blocking monoclonal antibody (10 µg/mL) evaluated by counting 10 fields per well under a phase contrast optical microscope after EDPs and AGVPGLGVG stimulations for 40 min. Data from one experiment, representative of three independent experiments, are shown. Results (mean ± S.E.M) were expressed as percentage of control (EDPs untreated cells). **p < 0.01, ***p < 0.001. **e** Real-time PCR analysis of RPSA 48 h after treatment with RPSA siRNA *vs* negative control siRNA-treated cells (Ctl siRNA). Results (mean ± S.E.M; *n* = 3) were expressed as the ratio of RPSA mRNA to EEF1a1 mRNA. **f** RPSA siRNA transfected HT-1080 cells incubated with TAMRA-AGVPGLGVG (AG9) at 5 × 10^−5^ M for 1 h at +4 °C. Confocal imaging was realised to study the TAMRA-AGVPGLVGV and RPSA localisations on HT-1080 cells. Scale bar: 10 µm. **g** RSPA expression was analysed in different cell types by western blot. Data from one experiment, representative of three independent experiments, are shown. **h** Quantitative evaluation of RPSA expression by different cell types. **i** Correlation between Δblebbing and ratio RPSA/actin in different cell types. Δblebbing = EDP-dependent blebbing – unstimulated blebbing. Linear regression test was performed for each analysis and the R and *p* values are indicated in the graph. **j** Optical section obtained by confocal microscopy of control HT-1080 cell and EDP-induced blebbing HT-1080 cell to analysis RPSA localisation; western blot for RPSA realised with 10 µg of HT-1080 cell extract and extracellular vesicle proteins. Scale bar: 10 µm

In absence of EDPs, HT-1080 cells presented mesenchymal cell morphology with lamellipodia and filopodia. RhoA, Rho-associated Coil-containing Protein Kinases (ROCK1, ROCK2), phosphorylated LIMK1/2, phosphorylated ERM (P-ERM) and cofilin presented a normal localisation in all cell body (Fig. 3d, e; Suppl.figure 6a–i; Suppl.table I). Calpain1 was predominantly localised in the nucleus. The actin and myosin light chain (MLC) networks were properly organised in the cytoplasm (video 5). During blebbing, EDPs triggered a translocation of these proteins into the blebs. Actin and MLC were disorganised (video 6). ROCK could be activated by either caspase-3 or RhoA.^40^ We studied whether ROCK was activated through the caspase-3-dependent pathway. Doxorubicin was used as a positive control to induce HT-1080 apoptosis characterised by cleaved caspase-3 expression and annexin-5 membrane binding (Fig. 3f). Consistently with trypan blue exclusion results (Suppl.figure 3), EDPs did not induce apoptosis as we did not detect cleaved caspase-3 expression (Fig. 3f), showing that ROCK activation was independent of the caspase-3 pathway. The cell membranes were annexin-5-negative, except the extracellular vesicles released during the cell contraction. Annexin-5 binding extracellular vesicles correspond to the released GFP-Hsp90 vesicles after cell retraction (Fig. 3f; video 7). These data indicated that EDP-dependent membrane blebbing was regulated by calcium influx, involved the Rho/ROCK/MLC pathway without apoptosis.

### Cell blebbing capacity is correlated with Hsp90 level

We previously shown that EDPs stimulate Hsp90 secretion in HT-1080 fibrosarcoma and MDA-MB-231 breast carcinoma cells.^5^ As Hsp90 was reported to localise in all blebs and extracellular vesicles, we decided to study its expression in different cell lines (Fig. 4a, b). Actin was used as a total protein loading control. No Hsp90 protein was detected in skin fibroblast and SK-MEL-28 melanoma cell culture media. HT-144 and A375 melanoma cell and HUH-7 hepatocarcinoma cell media contained Hsp90, but EDP treatment did not modify extracellular Hsp90 amounts. Adding EDPs to PLC-PRF-5 hepatocarcinoma cell, HT-29 colorectal adenocarcinoma cell, MDA-MB-231 and MDA-MB-435 breast adenocarcinoma cell, lung adenocarcinoma BZR cell and HT-1080 cell media significantly stimulated Hsp90 secretion: 1.3-fold, 3.3-fold, 2.6-fold, 3.6-fold, 2.2-fold and 5.0-fold, respectively.

Blebbing was studied in cell types with different cell migratory and invasive properties. EDPs increased blebbing in tumour cells previously reported to display highly invasive phenotypes, except for melanoma A375 cells that spontaneously undergo blebbing (Fig. 4c). As Hsp90 was induced by EDPs and reported to localise in blebs and extracellular vesicles, we decided to demonstrate a potential correlation between Hsp90 secretion and the blebbing phenotype in the different cell lines (Fig. 4c, d). No membrane bleb was detected for skin fibroblast and SK-MEL-28 melanoma cell after EDP treatment. With or without EDPs, HT-144 and A375 melanoma cell and HUH-7 hepatocarcinoma cell surface presented the same blebbing cell number. Adding EDPs to PLC-PRF-5 hepatocarcinoma cell, HT-29 colorectal adenocarcinoma cell, MDA-MB-231 and MDA-MB-435 breast adenocarcinoma cell, lung adenocarcinoma BZR cell and HT-1080 cell media significantly stimulated Hsp90 secretion: 1.3-fold, 3.3-fold, 2.6-fold, 3.6-fold, 2.2-fold and 5.0-fold, respectively (Fig. 4a, b). For the same cell lines, EDPs increased the cell surface blebbing: 2.6-fold, 3.9-fold, 2.2-fold, 3.7-fold, 3.9-fold and 3.1-fold, respectively. Interestingly, we showed a correlation between cell surface blebbing (Δblebbing) and Hsp90 secretion (ΔHsp90) in presence of EDPs (Fig. 4d).

### Blebbistatin and Y27632 inhibit EDP-stimulated Hsp90 and proteinase secretion

It is generally accepted that membrane blebbing requires RhoA and its main effector ROCK, which regulates the phosphorylation of MLC and the actomyosin contractility during the bleb life cycle.^25^ The requirement of MLC and ROCK for bleb formation was tested using the blebbistatin and Y27632, respectively (Fig. 4e and g). EDPs strongly stimulated the release of Hsp90, uPA, MMP-2 and MMP-9 by HT-1080 cells, as previously reported.^5^ Blebbistatin and Y27632 abolished EDP effects on Hsp90, MMP-9, MMP-2 and uPA secretion (Figs. 4f and h; Suppl.fig. 7). Our results demonstrate the involvement of MLC and ROCK in EPD-induced blebbing and proteases release and reinforce the correlation between blebbing and Hsp90 expression and release by HT-1080 cells.

### AGVPGLGVG and VGVAPG elastin peptides induce cell surface blebbing

EDPs obtained from purified insoluble elastin by organo-alkaline hydrolysis were composed of a mixture of different elastin peptides with an average molecular weight of 75 kDa. To identify which elastin peptide sequence could induce cell blebbing, VGVAPG, AGVPGLGVG and GRKRK synthetic peptides were tested. At different concentrations, VGVAPG and AGVPGLGVG peptides, but not GRKRK, induced bleb formation (Fig. 5).

### RPSA interacts with AGVPGLGVG elastin peptide and induce cell surface blebbing

ERC, galectin-3, αvβ3 and αvβ5 integrins were reported to bind elastin peptides. Lactose and chondroitin sulphate did not inhibit the blebbing, excluding ERC and galectin-3 (Suppl.table II). The use of αvβ5 blocking antibody had no significant effect on EDP-induced blebbing and αvβ3 blocking antibody blocked EDP-induced blebbing by 30% only. We decided to perform affinity chromatography on AGVPGLGVG and VGVAPG peptides. HT-1080 cell extracts were loaded on the columns and retained proteins were eluted using NaCl gradient. The eluted fractions were analysed by SDS-PAGE and silver staining. Three major bands of 67, 43 and 37 kDa were detected in the fraction eluted with 0.6 M NaCl (Fig. 6a). The 43 kDa protein was identified as RPSA by western blot analysis (Fig. 6b; Suppl.Fig. 8). This receptor was previously reported to bind elastin. The RPSA/AGVPGLGVG peptide colocalisation was confirmed by confocal microscopy on optical section (Fig. 6c). RPSA-blocking antibody abolished EDPs and AGVPGLGVG-induced blebbing (Fig. 6d). A RPSA siRNA approach was also used to prove RPSA involvement in EDP-blebbing. RPSA gene expression was decreased by 98% after RPSA siRNA transfection and protein expression was no more detectable by immunofluorescence (Fig. 6e). AGVPGLGVG was not able to bind HT-1080 cells after RPSA silencing as demonstrated by immunofluorescence studies (Fig. 6f). HT-1080 and MDA-MB-231 cells, which express high amount of RPSA, presented blebbing after EDP treatment, while skin fibroblast and SK-MEL-28 cells did not (Fig. 6g, h); the EDP-stimulated blebbing capacities was correlated to the RPSA expression (Fig. 6i). As Hsp90, MMP-2, MMP-14, αvβ3 integrin and actin, RPSA was found in the blebs and also in the extracellular vesicles at a higher concentration than in the HT-1080 cell extracts (Fig. 6j).

## Discussion

Matrikines like EDPs were shown to promote tumour cell invasion.^4,5,7^ The present study provides evidence that EDP treatment of tumour cells induces a switch from a mesenchymal cell phenotype to an amoeboid-like phenotype characterised by a highly dynamic membrane blebbing and by extracellular vesicle shedding. These findings may explain why EDPs favour tumour cell invasion and metastasis.^41–44^ Enhancement of elastin receptor expression was associated with poor clinical outcome in colon, gastric and breast carcinomas.^45^

Blebs are considered to be a hallmark of apoptosis. However, they are frequently observed during migration in three-dimensional cultures and in vivo. Loss of cell–cell contacts or changes in cell adhesion properties may promote bleb formation.^46^ Most of the experiments studying bleb formation were performed under serum-stimulating conditions in which blebbing was, in fact, dependent on the presence of FCS or lysophosphatidic acid.^47–49^ Cells undergo blebbing after exposure to substance P/neurokinin-1 receptor complex, H_2_O_2_, ATP and Pi.^50–55^ We demonstrated for the first time that ECM degradation products modify cancer cell morphology by generating a blebbing phenomenon in absence of FCS, leading to extracellular vesicle shedding. During tumour progression, elastin is cleaved by many proteinases secreted locally or circulating in serum. EDPs are released into the bloodstream according to the disease state and age of the patients.^56,57^ In addition to their well-known effects on tumour development by stimulating angiogenesis, tumour cell proliferation and proteinase secretion, our results show that EDPs increase cancer cell invasive capacities by inducing the bleb-associated amoeboid mode of invasion and the release of tumour extracellular vesicles.

To date, only two cell receptor have been described to mediate membrane blebbing: neurokinin-1 receptor and P2X7 receptor.^51,55^ Among the tested matrikines, the AGVPGLGVG peptide displays a strong effect on tumour cell blebbing through RPSA receptor binding. This receptor is over-expressed on the tumour cell surface in a variety of human carcinomas, and correlates with a higher proliferation rate and metastasis properties of malignant cells. RPSA-blocking antibody and EGCG treatment prevents EDP-induced blebbing (Suppl.Fig. 9; suppl.video E-H). The RPSA receptor is a remarkable, multifaceted protein whose functions extend from matrix adhesion to ribosome biogenesis. Its ability to engage extracellular laminin is further thought to contribute to cellular migration and invasion. Since 1989, by their common properties (same epitope, same molecular weight 67 kDa, laminin/elastin-binding, lectin properties), RPSA and ERC were considered as the same receptor.^14^ RPSA and ERC mediate cell migration and invasion but we demonstrate that only RPSA receptor induces plasma membrane blebbing lead to amoeboid cell motility and invasion. On other hand, by its dynamic blebbing, RPSA receptor participates to extracellular vesicle shedding and cell-to-cell communication. The correlation between RPSA expression and tumour aggressiveness proposes RPSA as a promising strong target in fight against cancer but also in prion diseases and Alzheimer’s disease.^18^ Therefore, by its multiple functions like maintenance of nuclear structures and translational processes, RPSA knockdown by siRNAs or CRISP-Cas9 approaches to elucidate whether or not targeting of RPSA significantly reduce tumour cell migration and invasion constitute a challenge. RPSA knockdown reduces tumour cell viability.^58^ Only the pharmacological approaches (green tea polyphenol EGCG) can overcome this difficulty.

After EDP/RPSA interaction, membrane bleb formation involves well already define signalling pathways like RhoA/ROCK/MLC cascade. Our results demonstrate that Y27632, a ROCK inhibitor, and blebbistatin, a myosin II inhibitor, inhibit the EDP-induced release of Hsp90, MMPs and uPA, showing the involvement of the RhoA/ROCK/MLC pathway. EDPs trigger a calcium influx that activates calpain. Calpain was reported to induce cytoskeletal protein degradation and actomyosin reorganisation. The breakdown of the actomyosin network associated with the hydrostatic pressure causes the membrane bleb formation. ROCK was previously reported to phosphorylate ERM that participates in actin cytoskeleton reorganisation and bleb formation.^59,60^ Taken together, our results suggest that EDP-induced blebbing involve the RhoA/ROCK pathway, the calpain activation, the phosphorylation of ERM proteins leading to the actin cytoskeletal reorganisation and bleb formation.

Extracellular vesicle were reported to contain a large amount of Hsp90 protein.^38^ We demonstrate a correlation between EDP-dependent bleb formation, shed extracellular vesicles and Hsp90 secretion. Highly invasive cancer cells undergo membrane bleb formation and a high Hsp90 release under EDP influence. Extracellular Hsp90 exacerbate cancer cell invasion as it participates in many proteinase activation and prevents their inactivation.^5,9,61^ Hsp90 secretion depends on yet unknown regulating mechanisms. We show in this paper that Hsp90 is strongly linked to bleb formation and extracellular vesicle shedding. The presence of Hsp90 in the shed extracellular vesicles could explain the high level of Hsp90 in plasma of cancer patients.

Besides the amoeboid migration movement, membrane blebbing allows the extracellular vesicle shedding in the cellular microenvironment.^30,62^ Blebs and shed extracellular vesicles presented common markers, like high amount of RPSA, phospho-ERM, Hsp90, αV integrin subunit and some proteinases. Tumour extracellular vesicles have been linked to Hsp90 secretion and tumour invasion via release of proteinases.^36,63^ Extracellular vesicles provide a large membrane surface for the activation of membrane-associated proteinases involved in extracellular matrix degradation and tissue invasion.^36^ The tumour cell release vesicles that exhibit membrane phosphatidylserine redistribution, one of the major events required of extracellular vesicle formation.

Taken together, our results demonstrate that matrikines induce cancer cell blebbing and extracellular vesicle release through RPSA binding, favouring dissemination, cell-to-cell communication and growth of cancer cells in metastatic sites. This process involves normal blebbing signalling pathway: calcium influx, RhoA/ROCK/MLC, calpain activation and ERM phosphorylation. Hsp90 and proteinase expression and release are increased after EDP treatment and is correlated with blebbing and Matrigel invasion.

## Supplementary information

Video G - Spinning disk microscopy of GFP-Hsp90 HT-1080 cell in presence of EGCG and in absence of EDPs

Video H - Spinning disk microscopy of GFP-Hsp90 HT-1080 cell in presence of EGCG and EDPs

S Fig 1 - EDPs stimulate cell membrane blebbing in 3D collagen matrix. GFP-Hsp90 transfected HT-1080 cells were seeded in a 3D collagen matrix in the presence of EDPs

S Fig 2 - 2D Time-lapse snapshots of blebbing HT-1080 cells in presence of EDPs at t0min and at t75min

S Fig 3 - Cell proliferation and cell survival tests

S Fig 4 - 2D Time-lapse snapshots of cell-to-cell communication between a blebbing cell and a mesenchymal cell in presence of EDPs

S Fig 5 - Extracellular vesicles were prepared from cell-conditioned medium by centrifugation and ultracentrifugation after 24h of incubation

S Fig 6 - Signalling pathway immunostaining quantifications and localizations using the ImageJ software

S Fig 7 - Blebbistatin and Y27632 inhibit EDP-stimulated blebbing, Hsp90 and proteinase secretions

Video 1. Spinning disk microscopy of a mesenchymal GFP-Hsp90 HT-1080 cell in absence of EDP

Video 2. Spinning disk microscopy of a blebbing GFP-Hsp90 HT-1080 cell in presence of EDPs

Video 3. Live videomicroscopy of the reversible blebbing in presence of EDPs

Video 4 - Spinning disk microscopy of cell-to-cell communication via shed extracellular vesicles in presence of EDPs

Video 5 - Spinning disk microscopy of mesenchymal mCherry-MLC HT-1080 cells in absence of EDP

Video 6 - Spinning disk microscopy of blebbing mCherry-MLC HT-1080 cells in presence of EDPs

Video 7 - Spinning disk microscopy of blebbing GFP-Hsp90 HT-1080 cells and shed microsicles in presence of EDPs

S Table 1. Immunostaining quantification and localization in HT-1080 cells using ImageJ plugin

S Table 2. Blebbing quantification in HT-1080 cells in presence of different elastin receptor inhibitors

S Fig 8 - Identification of the RPSA protein as the VGVAPG receptor by affinity chromatography

S Fig 9 - EGCG inhibits EDP-stimulated blebbing

Video A - Spinning disk microscopy of a mesenchymal GFP-Hsp90 HT-1080 cell in absence of EDP

Video B - Spinning disk microscopy of blebbing GFP-Hsp90 HT-1080 cell in presence of EDPs

Video C - Live videomicroscopy of blebbing HT-1080 cells in presence of EDPs

Video D - Live videomicroscopy of cell-to-cell communication via shed microvesicle in presence of EDPs

Video E - Spinning disk microscopy of GFP-Hsp90 HT-1080 cell in absence of EDPs

Video F - Spinning disk microscopy of GFP-Hsp90 HT-1080 cell in presence of EDPs

## Data Availability

Material, data and associated protocols are available to readers upon request.
