# Supplementary figures and images for: Tumour cell blebbing and extracellular vesicle shedding: key role of matrikines and ribosomal protein SA
Source: Br J Cancer. 2019 Feb 11;120(4):453–65. doi: 10.1038/s41416-019-0382-0 (PMC6461924; doi:10.1038/s41416-019-0382-0)

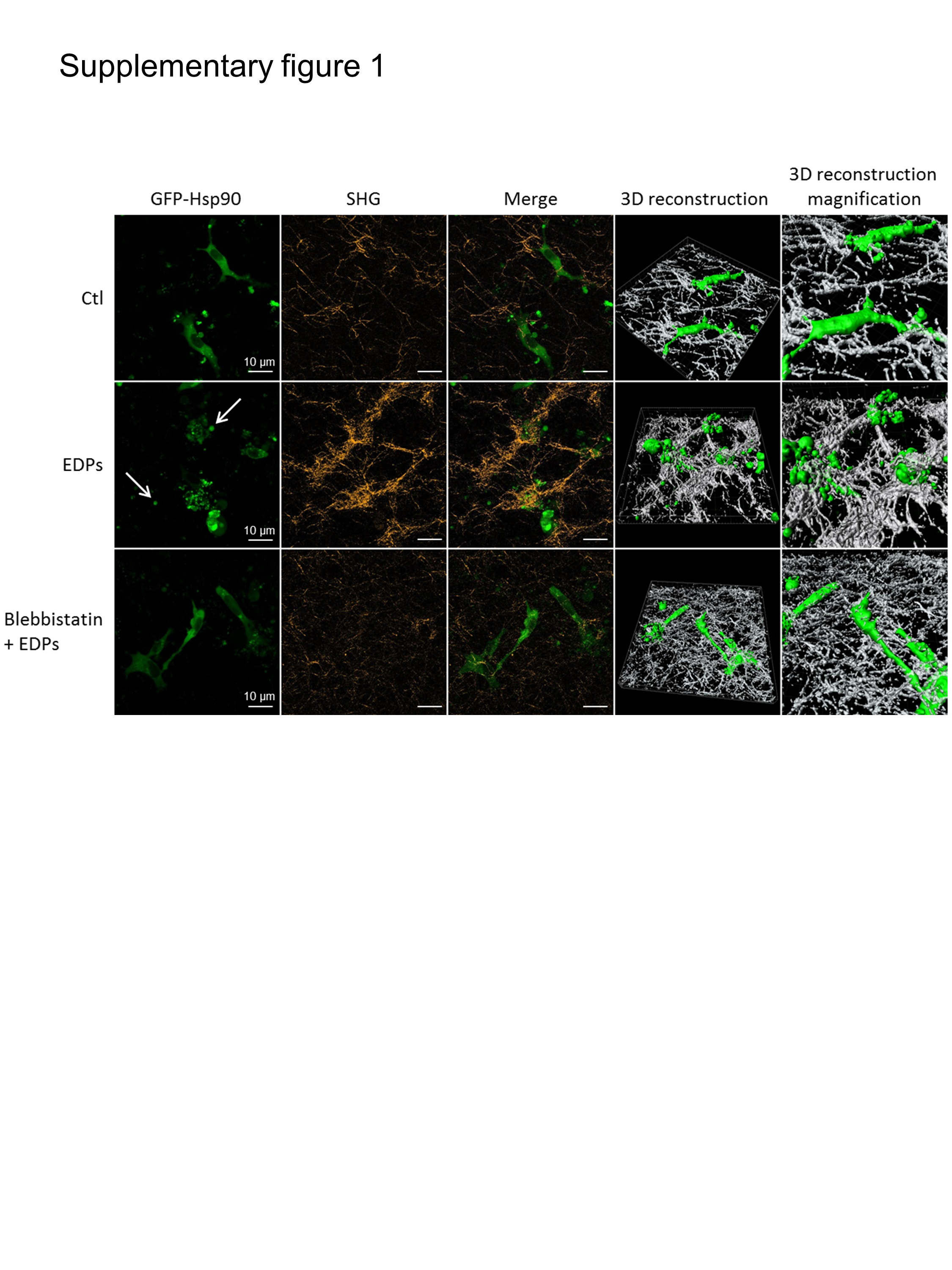

Supplement: Supplementary file 3 — S Fig 1 - EDPs stimulate cell membrane blebbing in 3D collagen matrix. GFP-Hsp90 transfected HT-1080 cells were seeded in a 3D collagen matrix in the presence of EDPs [file 41416_2019_382_MOESM3_ESM.tif]

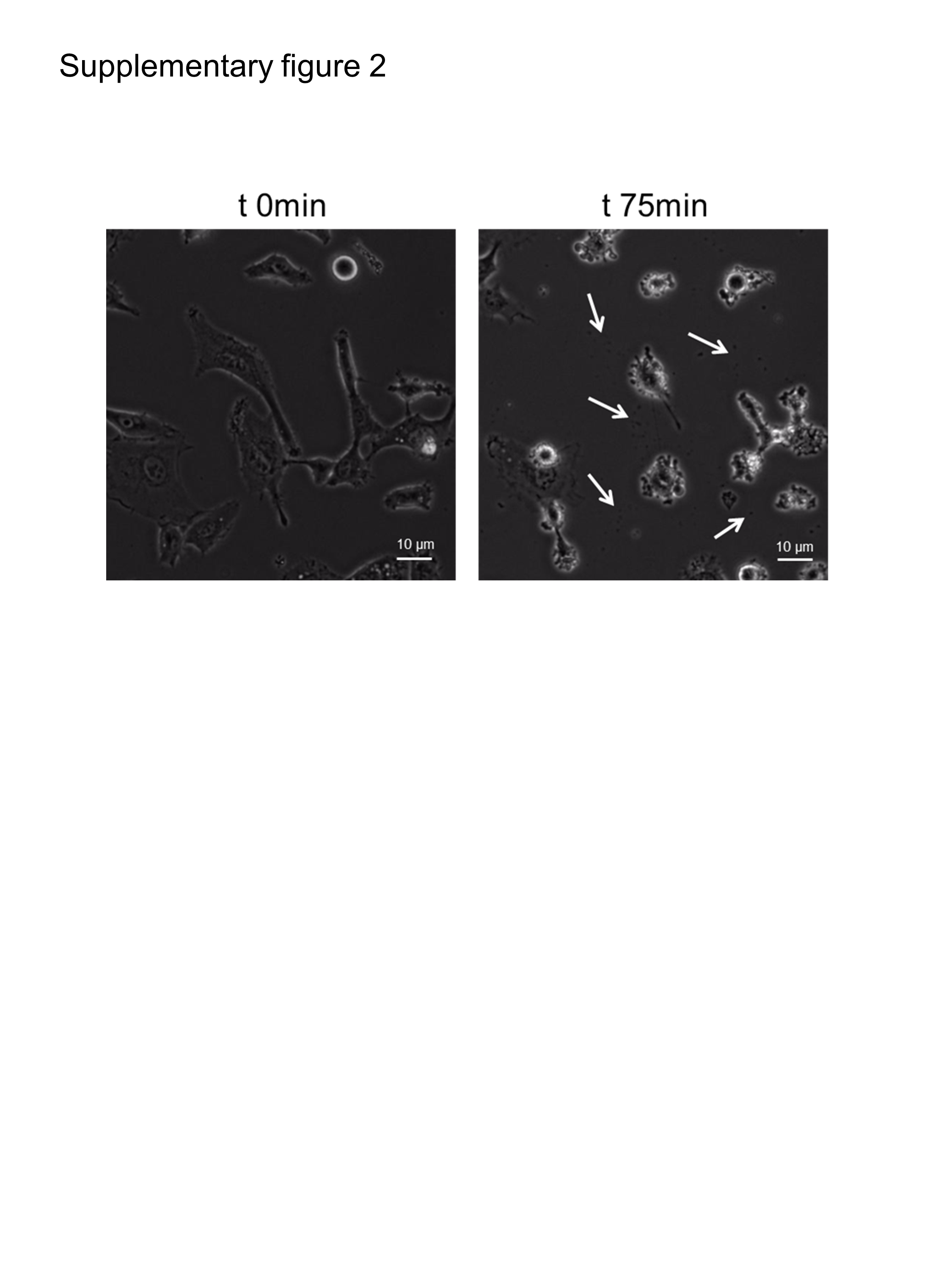

Supplement: Supplementary file 4 — S Fig 2 - 2D Time-lapse snapshots of blebbing HT-1080 cells in presence of EDPs at t0min and at t75min [file 41416_2019_382_MOESM4_ESM.tif]

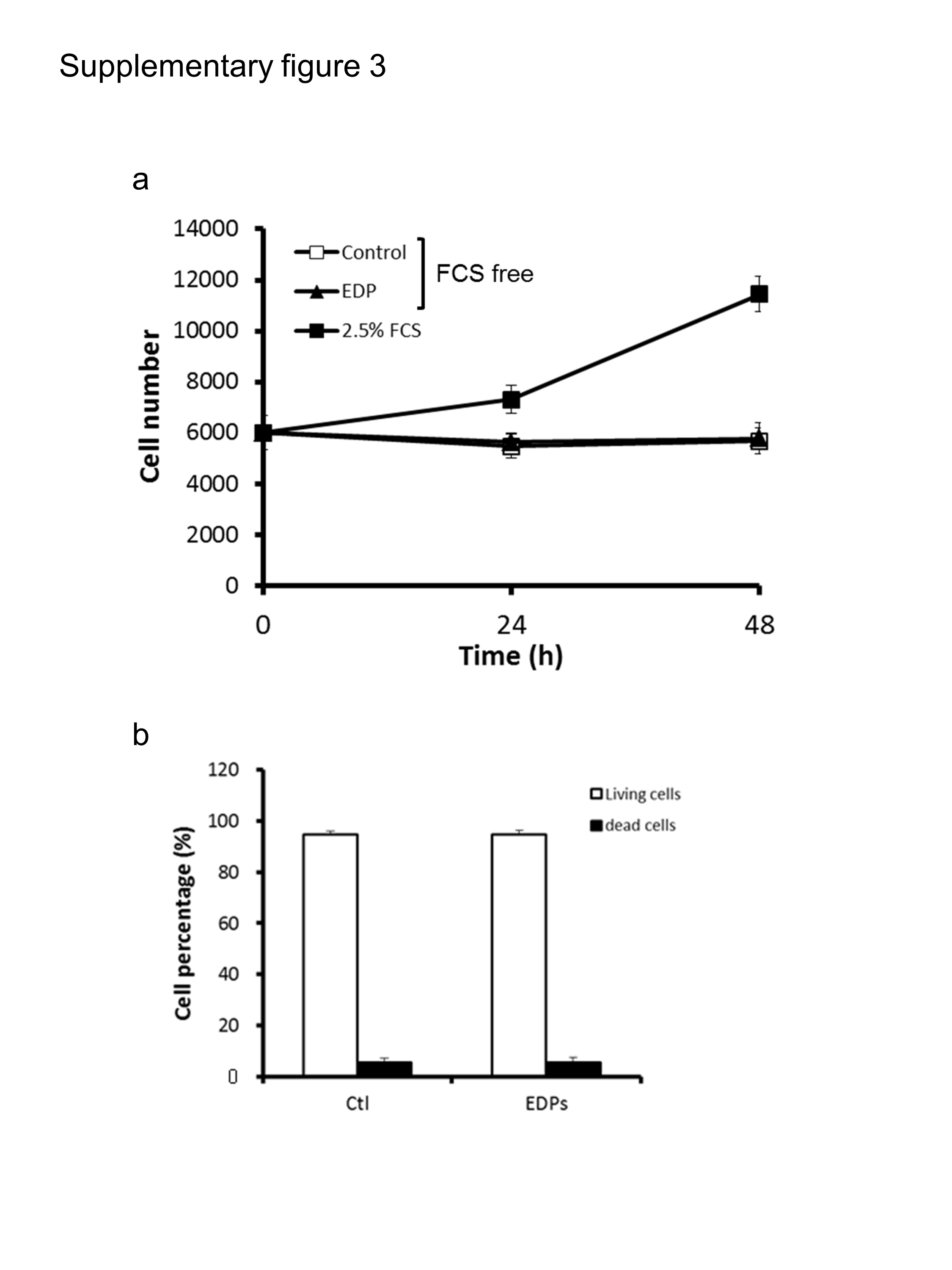

Supplement: Supplementary file 5 — S Fig 3 - Cell proliferation and cell survival tests [file 41416_2019_382_MOESM5_ESM.tif]

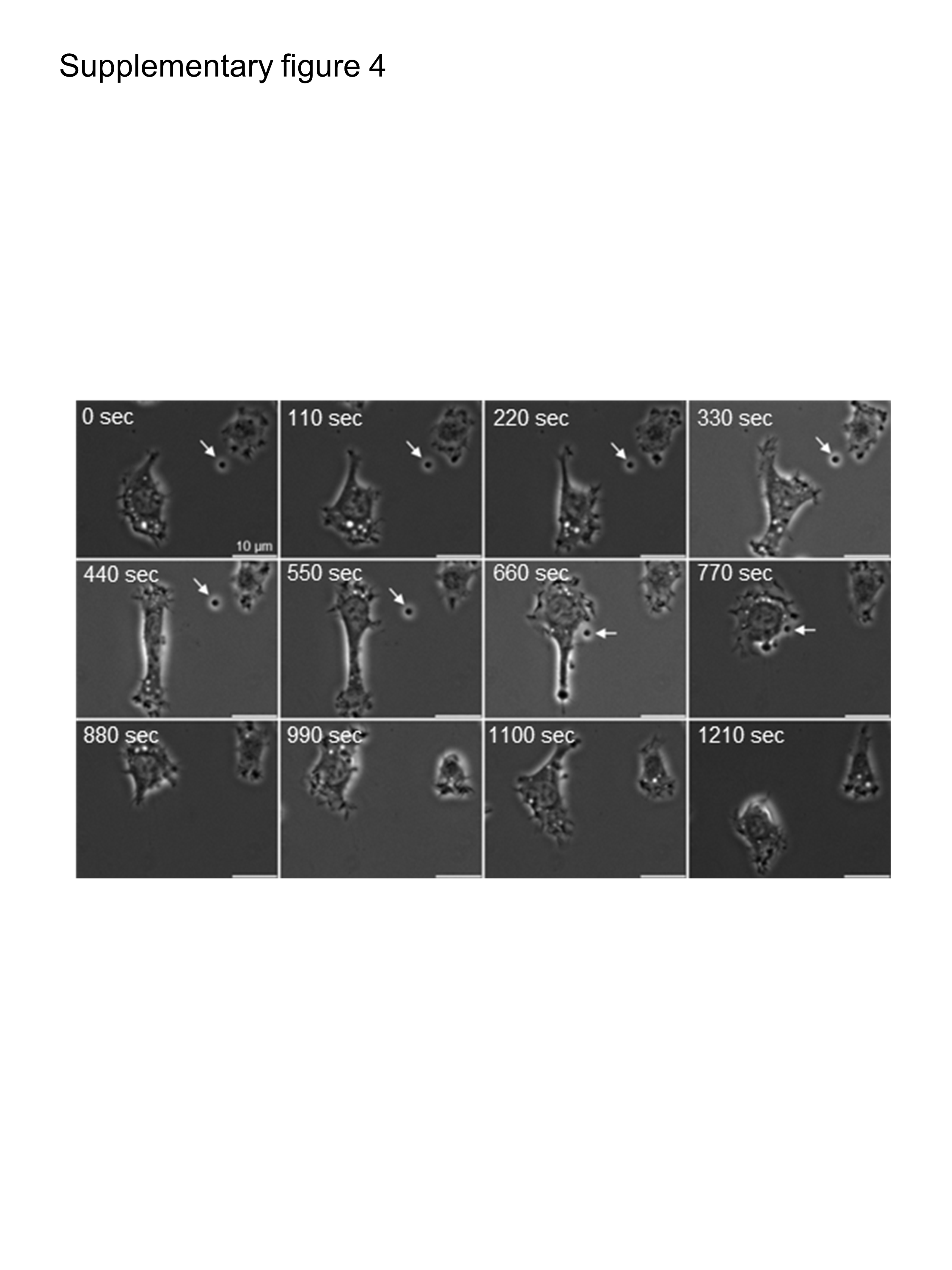

Supplement: Supplementary file 6 — S Fig 4 - 2D Time-lapse snapshots of cell-to-cell communication between a blebbing cell and a mesenchymal cell in presence of EDPs [file 41416_2019_382_MOESM6_ESM.tif]

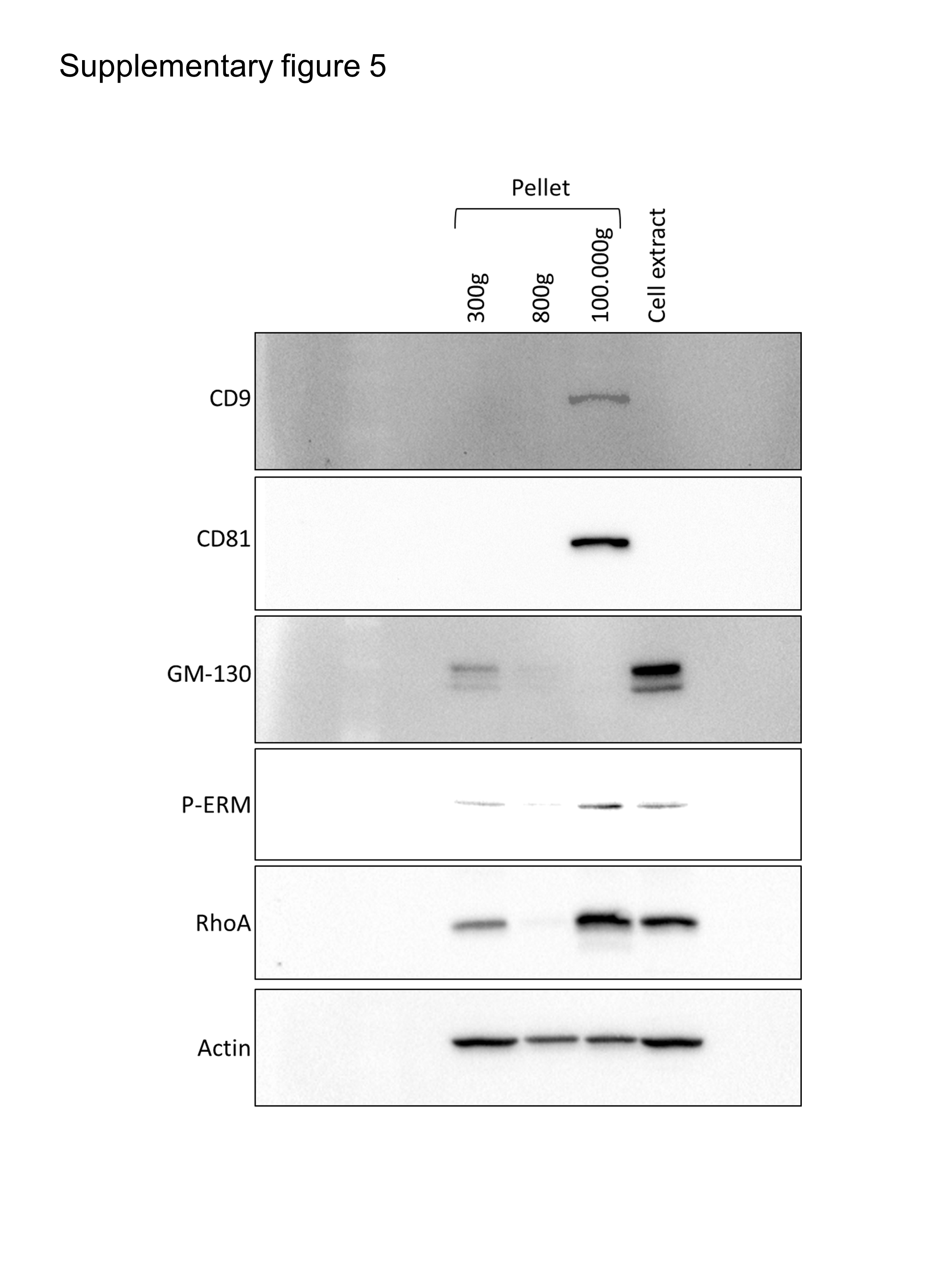

Supplement: Supplementary file 7 — S Fig 5 - Extracellular vesicles were prepared from cell-conditioned medium by centrifugation and ultracentrifugation after 24h of incubation [file 41416_2019_382_MOESM7_ESM.tif]

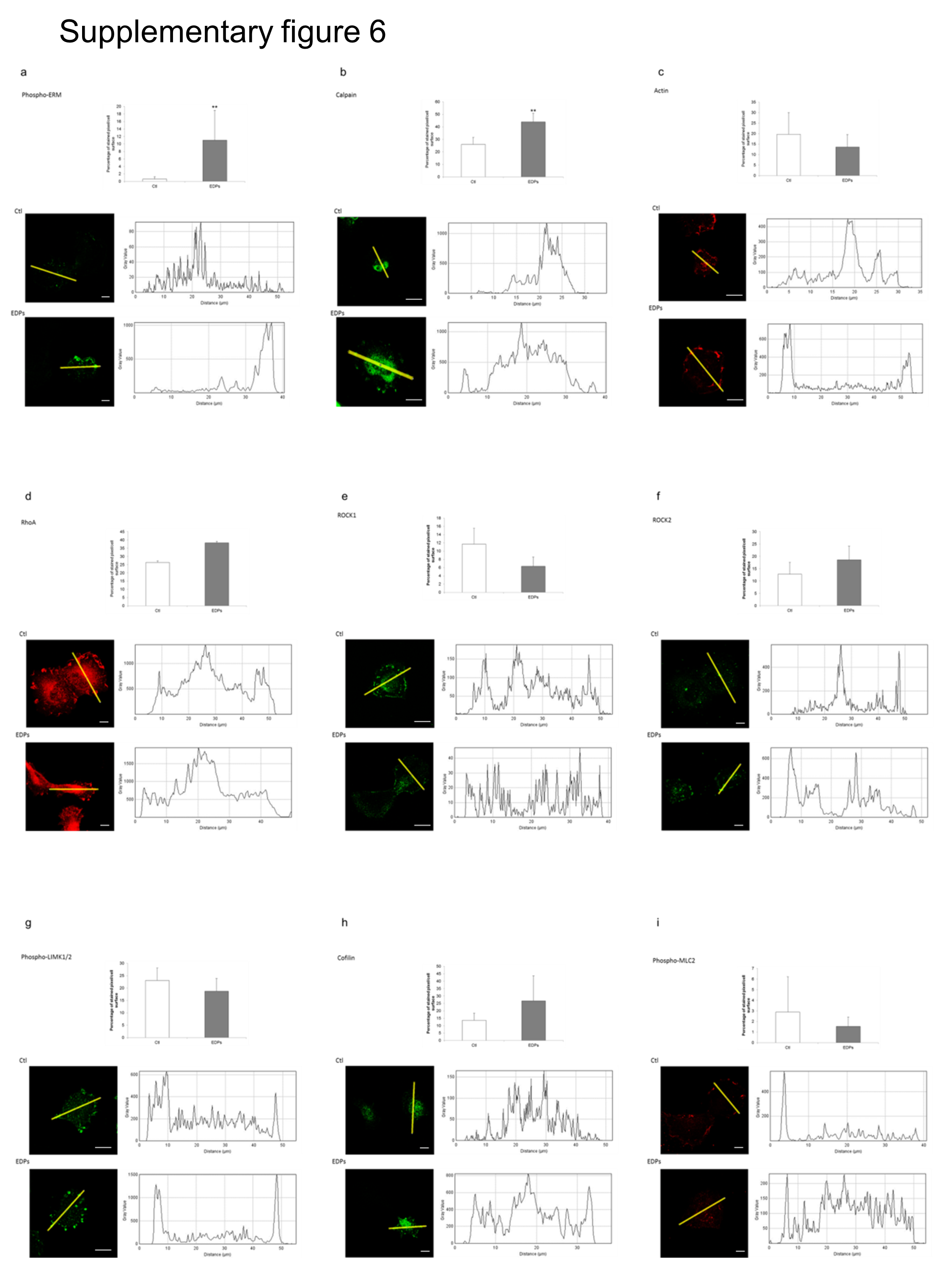

Supplement: Supplementary file 8 — S Fig 6 - Signalling pathway immunostaining quantifications and localizations using the ImageJ software [file 41416_2019_382_MOESM8_ESM.tif]

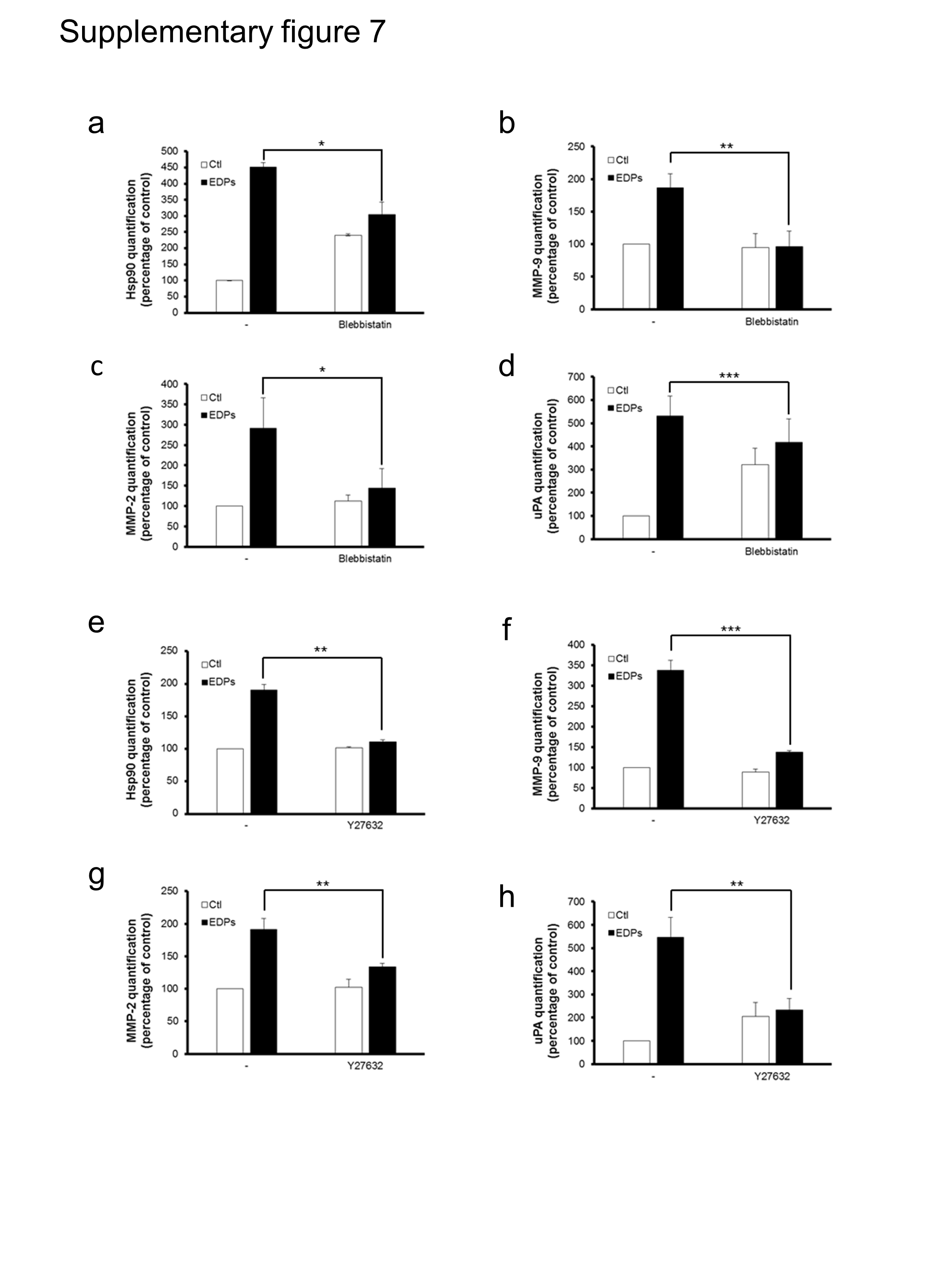

Supplement: Supplementary file 9 — S Fig 7 - Blebbistatin and Y27632 inhibit EDP-stimulated blebbing, Hsp90 and proteinase secretions [file 41416_2019_382_MOESM9_ESM.tif]

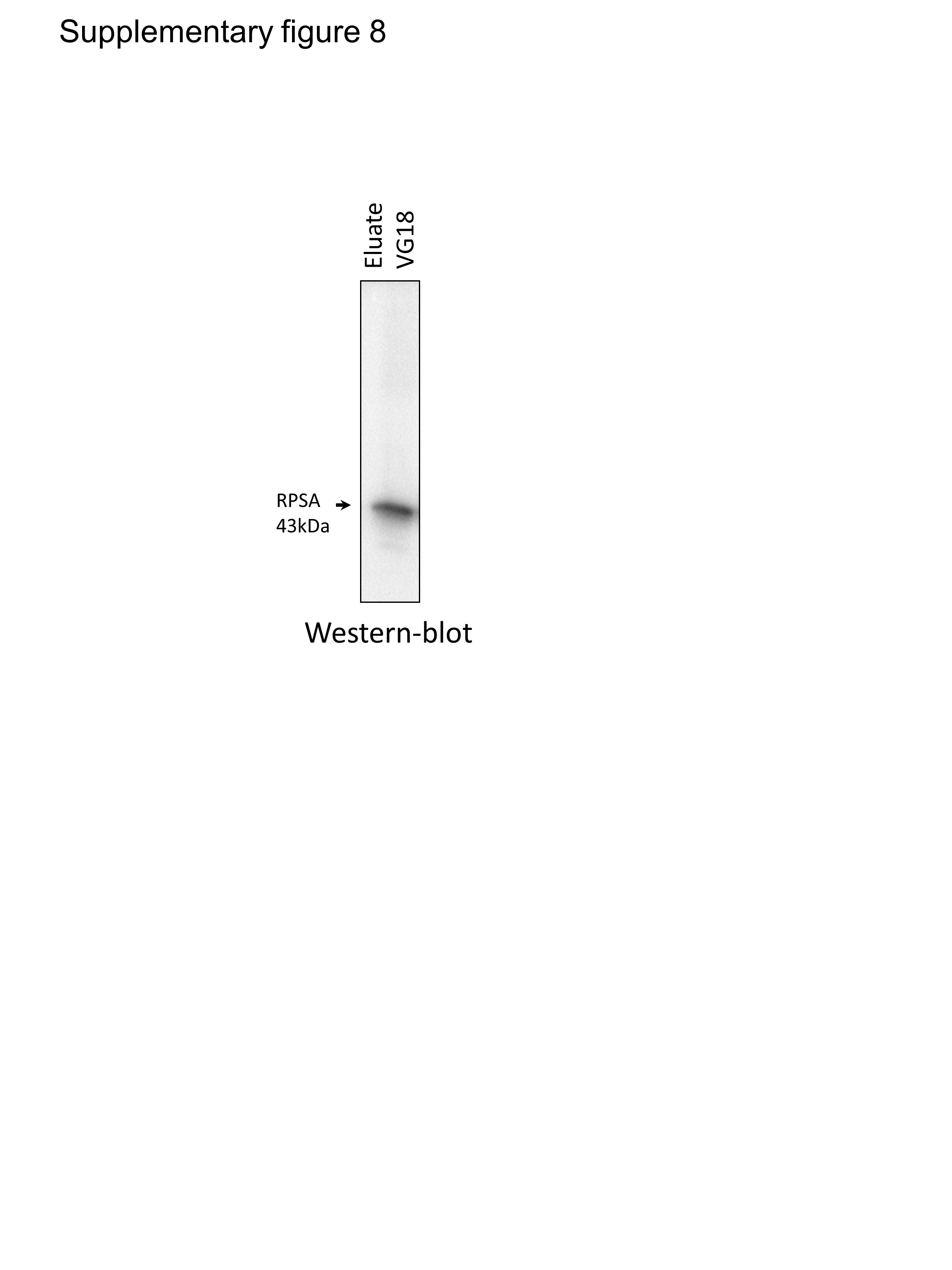

Supplement: Supplementary file 19 — S Fig 8 - Identification of the RPSA protein as the VGVAPG receptor by affinity chromatography [file 41416_2019_382_MOESM19_ESM.tif]

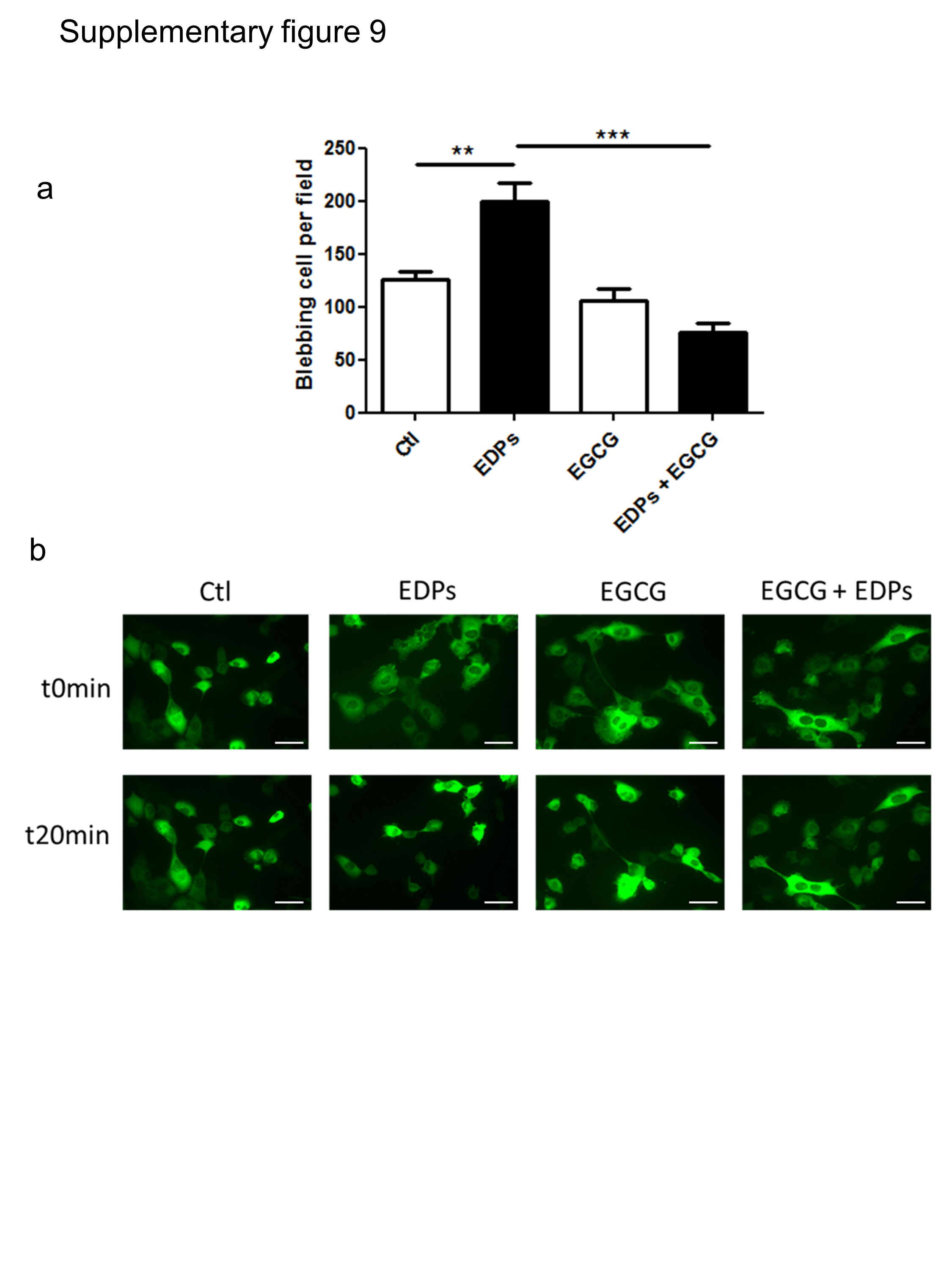

Supplement: Supplementary file 20 — S Fig 9 - EGCG inhibits EDP-stimulated blebbing [file 41416_2019_382_MOESM20_ESM.tif]
